# The first high-resolution meteorological forcing dataset for land process studies over China

**DOI:** 10.1038/s41597-020-0369-y

**Published:** 2020-01-21

**Authors:** Jie He, Kun Yang, Wenjun Tang, Hui Lu, Jun Qin, Yingying Chen, Xin Li

**Affiliations:** 1 Ministry of Education Key Laboratory for Earth System Modeling, Department of Earth System Science, Tsinghua University, Beijing, 100084 China; 2 Center for Excellence in Tibetan Plateau Earth Sciences, Institute of Tibetan Plateau Research, Chinese Academy of Sciences, Beijing, 100101 China; 3 Center of Earth Observation and Big Data Analysis for Three Poles, Institute of Tibetan Plateau Research, Chinese Academy of Sciences, Beijing, 100101 China

**Keywords:** Environmental sciences, Hydrology

## Abstract

The China Meteorological Forcing Dataset (CMFD) is the first high spatial-temporal resolution gridded near-surface meteorological dataset developed specifically for studies of land surface processes in China. The dataset was made through fusion of remote sensing products, reanalysis datasets and *in-situ* station data. Its record begins in January 1979 and is ongoing (currently up to December 2018) with a temporal resolution of three hours and a spatial resolution of 0.1°. Seven near-surface meteorological elements are provided in the CMFD, including 2-meter air temperature, surface pressure, and specific humidity, 10-meter wind speed, downward shortwave radiation, downward longwave radiation and precipitation rate. Validations against observations measured at independent stations show that the CMFD is of superior quality than the GLDAS (Global Land Data Assimilation System); this is because a larger number of stations are used to generate the CMFD than are utilised in the GLDAS. Due to its continuous temporal coverage and consistent quality, the CMFD is one of the most widely-used climate datasets for China.

## Abstract (summary)

**Table:** 

Measurement(s)	temperature • pressure • humidity • atmospheric wind speed • radiation • precipitation process
Technology Type(s)	digital curation
Factor Type(s)	geographic location • time
Sample Characteristic - Environment	climate system
Sample Characteristic - Location	China

Machine-accessible metadata file describing the reported data: 10.6084/m9.figshare.11558439

## Background & Summary

Land, hydrological and ecosystem models all require the input of gridded near-surface meteorological datasets, called “forcing data”. Accurate and high-resolution forcing data can help improve the outcome of these models, hence, high-quality forcing data are always desired by these scientific communities. To meet this demand, efforts were made during the 2000s to develop global-scale datasets specially for land surface and hydrology research, e.g. Princeton University’s Global Land Surface Model Data^1,2^ and the Global Land Data Assimilation System (GLDAS)^3^. Meanwhile, remote sensing datasets obtained from some earth-observing satellites, like the Tropical Rainfall Measuring Mission (TRMM) precipitation rate dataset^4^, and remote sensing-derived data products such as Climate Prediction Center (CPC) Merged Analysis of Precipitation (CMAP)^5–8^, provided more choices for data on particular variables to land surface and hydrology researchers.

Beside these global datasets, a few datasets have been developed to improve the accuracy of meteorological data on regional scale; e.g., the North American Land Data Assimilation System (NLDAS)^9–11^, the Canadian Land Data Assimilation System (CaLDAS)^12^, and the European Land Data Assimilation System (ELDAS)^13^.

In China, the global datasets have been widely used for more than a decade, yet, there is much room for improvement in their representation over China itself. On the one hand, the spatial resolution of the stated datasets is not high enough to depict complex weather/climate patterns over the mountainous terrain in mid-west China. On the other, the generation of such datasets necessitates ground-based observations as inputs. The more stations the input observation dataset contains the better forcing dataset is expected to be. However, observations at only a small fraction of weather stations from the China Meteorological Administration (CMA) are shared world-wide via the Global Telecommunication System (GTS), which prevents data developers from improving their dataset quality over China.

Since the beginning of this century, CMA has begun to share its observational datasets through the China Meteorological Data Service Center (CMDC), providing an opportunity to improve the accuracy of existing forcing datasets in China. Observed near-surface meteorological data at about 700 weather stations in China are routinely publicized with a lag of about three months, a period taken for data compilation and quality control.

Since 2008, we have used this precious data-sharing opportunity to develop the first high-resolution meteorological dataset, called the China Meteorological Forcing Dataset (CMFD)^14,15^ and based on the released CMA data. The CMFD is a gridded dataset from January 1979 to present (currently December 2018), with a spatial resolution of 0.1° and a temporal resolution of three hours. Its grid points are evenly distributed in a region of 70–140 °E, 15–55 °N, though only the grid points in China’s mainland area have valid values. The CMFD contains all seven near-surface meteorological elements required by land modelling, including 2-meter air temperature, surface pressure, and specific humidity, 10-meter wind speed, downward shortwave radiation, downward longwave radiation, and precipitation rate. The physical definitions of these variables are listed in Table 1.

**Table 1:** Definitions of variables in the CMFD.

Variables	Variable name	Unit	Physical meaning
Temperature	temp	K	Instantaneous near surface (2 m) air temperature.
Pressure	pres	Pa	Instantaneous near surface (2 m) air pressure.
Specific humidity	shum	kg kg^−1^	Instantaneous near surface (2 m) air specific humidity.
Wind speed	wind	m s^−1^	Instantaneous near surface (10 m) wind speed.
Downward shortwave radiation	srad	W m^−2^	3-hourly mean (from −1.5 hr to +1.5 hr) surface downward shortwave radiation.
Downward longwave radiation	lrad	W m^−2^	3-hourly mean (from −1.5 hr to +1.5 hr) surface downward longwave radiation.
Precipitation rate	prec	mm hr^−1^	3-hourly mean (from −3.0 hr to 0.0 hr) precipitation rate.

Meanwhile, two other institutes started developed high-resolution meteorological datasets in China. One is the CMA Meteorological Information Center, which is developing the High-Resolution CMA Land Data Assimilation System (HRCLDAS) product^16^. It uses as much observational data as possible^17^, but provides data only from 2008 onwards. The other is the Beijing Normal University (BNU), which offers a near-surface meteorological dataset up to 2010^18^, though there are no more recent data available.

The key features of the CMFD are its long length, stability, and continuity, characteristics that are continuously emphasized in the development of the dataset. Although this dataset was intended to provide a better dataset to drive a variety of terrestrial models over China, it has already been applied to much broader fields. These include fields such as climate model validation, climate zone classification, scheduling of crop planting and many other terrestrial research fields, and the CMFD has become one of most sought-out datasets by the land surface research community in China.

## Methods

### Input data

**Fig. 1:**
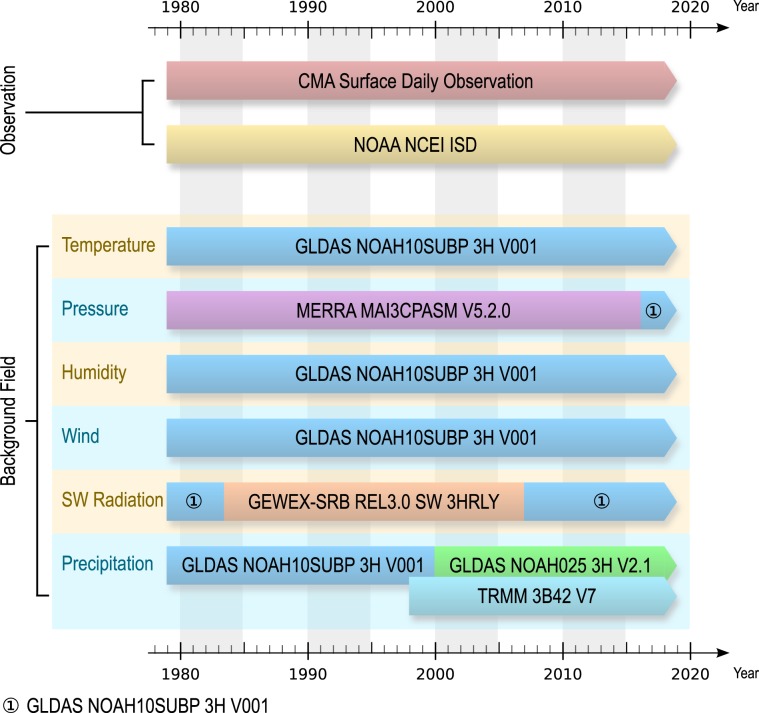
Datasets used to create the CMFD. Note that different combinations of background field datasets are chosen to create different variables. In particular, three datasets are used to create precipitation data; the TRMM 3B42 dataset is used as the first-priority data, while the remaining two, GLDAS NOAH10SUBP 3H and GLDAS NOAH025 3H,are used as the complements of the TRMM 3B42 dataset.

The CMFD was made through fusion of ground-based observations with several gridded datasets from remote sensing and reanalysis (Fig. 1). The ground-based observations used in this study come from two data sources: those acquired from CMA’s CMDC are daily data from approximately 700 stations, while those from the National Oceanic and Atmospheric Administration (NOAA)’s National Centers for Environmental Information (NCEI) are sub-daily data with only 300–400 stations available over China for most years^19^. They are the backbone of the CMFD. Because both datasets are CMA weather station data, we will not distinguish them, and collectively call them CMA data throughout the remainder of this paper. The gridded reanalysis/remote sensing data used in this study are GLDAS NOAH10SUBP 3H, GLDAS NOAH025 3H, Modern Era Retrospective-Analysis for Research and Applications (MERRA) MAI3CPASM 5.2.0^20^, Global Energy and Water Exchanges – Surface Radiation Budget (GEWEX-SRB) REL3.0 SW 3HRLY^21^, and TRMM 3B42 v7, all of which have a temporal resolution of three hours. The spatial resolution is 0.25° for GLDAS NOAH025 3H and TRMM 3B42 v7, and 1.0° for the remaining datasets.

The CMFD is expected to cover the years from 1979 to present and the mainland area of China. However, some input gridded datasets do not cover the entire time period (e.g. GEWEX-SRB REL3.0 SW 3HRLY) or the entire area (e.g. TRMM 3B42 v7). As a result, some additional datasets are selected as a complement when and where the first-priority datasets do not have full coverage. An example is the producing of precipitation data. The TRMM 3B42 v7 starts from 1998 and covers only the area between 40°S to 40°N, though it is used as a first-priority dataset due to its higher accuracy than other candidates. To expand the spatial-temporal coverage of this input data, we use GLDAS NOAH025 3H as the complement in the area to the north of 40°N. Yet, GLDAS NOAH025 3H starts from the year 2000, hence a third dataset, GLDAS NOAH10SUBP 3H, is introduced to make up when and where the former two datasets cannot cover. The datasets used for generating the data of each variable are shown in Fig. 1. A possible error may come from the discontinuity among the datasets with different data sources, but this error can be alleviated through fusion with the continuous station data.

Attention must be paid to the CMA data quality before conducting data fusion. CMA had carried out data quality control before the CMA data were released; however, we found some unexpected errors in the data, which may cause outliers in our product. Therefore, we have spent considerable time on the data quality control.

### An overview of algorithms for generating CMFD

Although observations at CMA weather stations are reliable, these stations are sparse in western China. As a result, it is inappropriate to create a gridded forcing dataset in western China by simply interpolating the station observations into grid points. On the contrary, reanalysis/remote sensing datasets are spatio-temporally consistent, but they usually have substantial systematic biases. Thus, merging these two kinds of datasets can mutually compensate for their deficiencies and result in a better dataset.

The algorithm for merging observational data and background data is based on the empirical knowledge that either their difference or their ratio is smoother in space than the data itself. In other words, the representation of ground-based observations is quite limited, especially in regions with complex terrain. So, a direct interpolation of the variable value can cause larger errors than the interpolation of the difference or the ratio between the station data and the background data. The latter is a basic algorithm for spatial interpolation.

Different data-generating algorithms were designed for the seven variables in the CMFD. Algorithms for temperature, pressure, specific humidity and wind speed are quite similar, so they are classified into one group and will be described as a whole. The algorithms for the remaining three variables, shortwave radiation, longwave radiation and precipitation rate, will be described separately.

### Temperature, pressure, specific humidity and wind speed

**Fig. 2:**
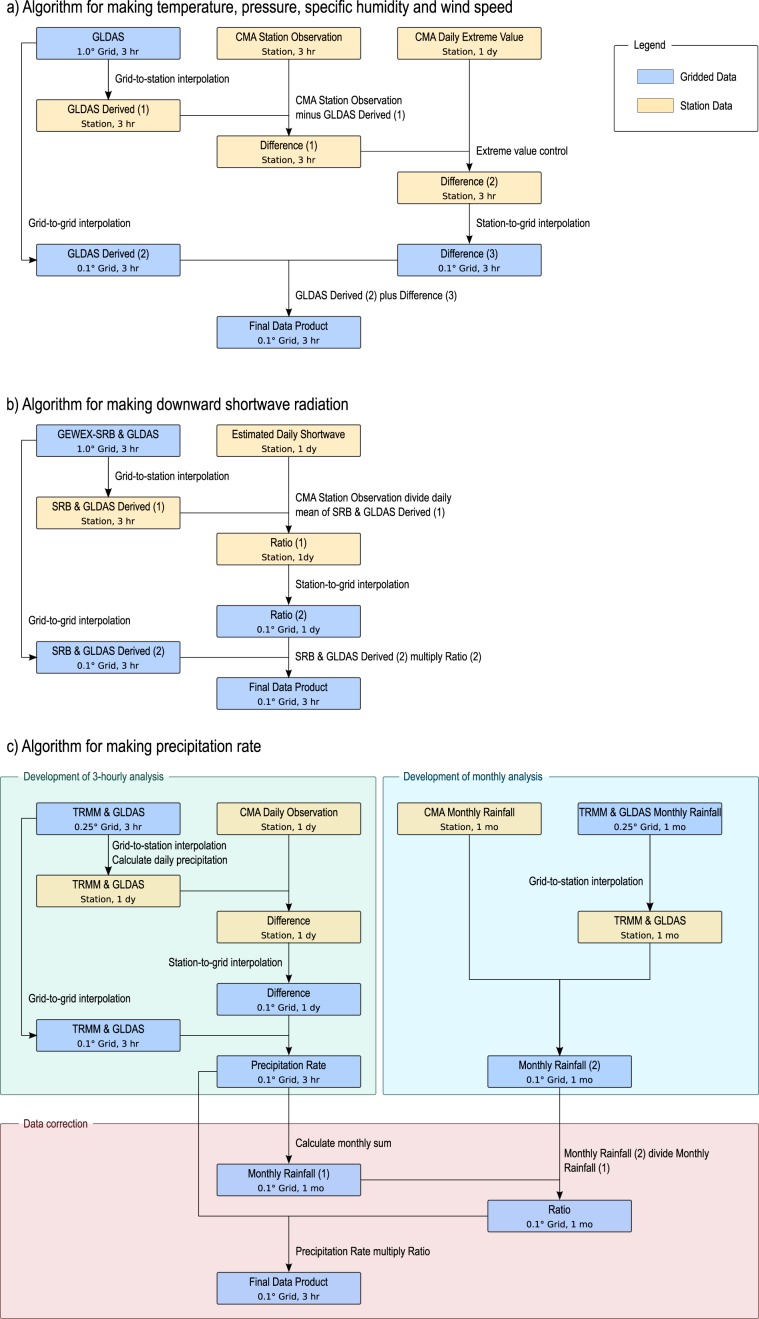
Algorithms for generating data for different variables in the CMFD. The algorithms for generating temperature, pressure, specific humidity and wind speed are quite similar so they are summarized as (**a**), and algorithms for making downward shortwave radiation and precipitation are depicted as (**b**,**c**), respectively.

The core of the algorithms for temperature, pressure, specific humidity and wind speed is summarized in Fig. 2a. The five main steps in this algorithm are listed as follows.

1. Interpolate 3-hourly gridded background data (GLDAS or MERRA) at the location of each CMA weather station.
2. Subtract interpolated background data from step (1) by 3-hourly observations at each station, obtaining the discrepancies between these two kinds of datasets.
3. Interpolate these discrepancies from stations to 0.1° grid points using ANUSPLIN software.
4. Remap the background data from its resolution to 0.1° grid.
5. Add the gridded discrepancies in step (3) to the output of step (4), to get the corrected data product.

Some additional treatments are applied to the interpolating process of particular variables. We introduced high-resolution elevation data in the interpolation of air temperature and pressure, as both of them are sensitive to altitude. Specifically, we first calculated sea-level temperature and pressure for the observational and reanalysis data respectively, then merged the two kinds of data according to the above algorithm. Finally, we calculated the air temperature and pressure at the land-surface altitude using high-resolution terrain elevation data. Likewise, in the atmosphere, specific humidity generally reduces with height, however, it does not have a well-established mathematical relationship with height like that of air temperature and pressure. Here we use relative humidity when doing spatial interpolation, because relative humidity is less sensitive to height than specific humidity, and thus is “smoother” is space.

### Downward shortwave radiation

The procedure for generating downward shortwave radiation data (Fig. 2b) is different to that for generating temperature, pressure, specific humidity, and wind speed. There are few stations that measure downward shortwave radiation in China. Therefore, we estimated the daily shortwave radiation using a hybrid model^22,23^ from station-observed daily sunshine duration and 2-meter air temperature, pressure, and humidity data. The estimated radiation data have been shown to be reliable and are thus used as a surrogate for observational data. However, the estimated shortwave radiation is daily data. In order to obtain the diurnal cycle of the radiation, we interpolate the ratio instead of the difference between the estimated radiation and the background data (GLDAS or GEWEX-SRB), and the interpolated ratio is then used to adjust background gridded data at every 3-hourly timestep. A merit of this algorithm is to ensure that the downward shortwave radiation is exactly zero during night time. The procedure for generating downward shortwave radiation data is listed as follows.

1. Interpolate 3-hourly gridded background data (GLDAS or GEWEX-SRB) at the location of each CMA weather stations.
2. Estimate the daily mean downward shortwave radiation with the hybrid model based on weather station data.
3. Calculate the ratio of the data from step (2) and the daily mean downward shortwave radiation from step (1).
4. Interpolate the ratio from stations to 0.1° grid points and obtain the gridded ratio.
5. Remap the background data from its resolution to 0.1° grid.
6. Multiply the gridded ratio in step (4) with the 3-hourly output of step (5), to get the corrected data product.

### Downward longwave radiation

The downward longwave radiation depends strongly on near-surface air temperature and vapour pressure as well as sky emissivity. The clear-sky emissivity is calculated using a semi-empirical formula given by Brutsaert^24^. The all-sky emissivity depends on cloud condition. Crawford and Duchon (1999, hereafter CD99)^25^ considered the cloud-sky as a blackbody and the all-sky emissivity as cloud fraction-weighted, with the cloud fraction being calculated from all-sky and clear-sky downward shortwave radiation.

Using the CD99 model, the downward longwave radiation is estimated directly from gridded data of 2-meter temperature, surface pressure, specific humidity, and downward shortwave radiation data that are obtained above.

### Precipitation rate

The algorithm for precipitation is more complex than that for other variables, mainly because precipitation has high spatial heterogeneity. Without observations from densely-spaced weather stations, common mathematical interpolation methods are unable to yield realistic distributions of precipitation at high spatiotemporal resolution, and an interpolation of precipitation similar to that of temperature will lead to negative values in sub-daily interpolated precipitation data. These negative values would then need to be adjusted to be zero. This adjustment may cause significant positive biases in monthly or yearly accumulated precipitation. For example, the yearly accumulated precipitation is often tens of millimeters higher than observations. As a result, these positive biases must be handled in the algorithm.

The basic idea to suppress the positive biases is to interpolate precipitation on sub-daily and monthly scales, respectively, and then adjust the sub-daily interpolated values according to the monthly interpolated values. Because the spatial distribution of precipitation is much smoother on a monthly scale than it is on a sub-daily scale, the monthly interpolation is able to produce more reliable results than the sub-daily interpolation. Therefore, the sub-daily interpolated values are proportionally adjusted so that the monthly values accumulated from the sub-daily results are identical to the monthly interpolated values. Therefore, the algorithm consists of the three steps that are distinguished by the three colored blocks in Fig. 2c.

First, we made a 0.1-degree, 3-hourly precipitation dataset through the interpolation algorithm similar to that of the temperature; the interpolation uses observational and 3-hourly gridded precipitation background data as inputs (see the upper-left block in Fig. 2c). The observations include 3-hourly data directly observed or downscaled from observed daily data with the aid of 3-hourly background data. Negative precipitation values of the interpolation are set to zero.

Second, a 0.1-degree, monthly precipitation dataset was made using the same interpolation algorithm, but using monthly observational and gridded precipitation background data as inputs (see the upper-right block in Fig. 2c).

Third, the ratio of this monthly precipitation data and the monthly precipitation derived from the 3-hourly precipitation dataset is calculated on each grid cell for each month. The ratio is then used as a correction factor to multiply the 3-hourly precipitation values on the grid for the month, which yields the final precipitation product (see the lower block in Fig. 2c).

## Data Records

The complete CMFD datasets are available online at figshare^14^ and the National Tibetan Plateau Data Center^15^. All CMFD data are stored in Network Common Data Form (NetCDF) files. Alongside the standard 3-hourly product, data of daily mean, monthly mean, yearly mean, and long-term climatological mean are also provided to users. Data files with different temporal resolutions are stored in separate directories, e.g. Data_forcing_03hr_010deg, Data_forcing_01dy_010deg, Data_forcing_01mo_010deg, and so forth. The naming convention for each type of data file is similar. As an example, the standard 3-hourly, 0.1° product’s data are arranged in separate files for each month and each variable. The naming convention follows the template VVVV_CMFD_Vvvvv_B-01_TTTT_SSSdeg_YYYYMM.nc (e.g. temp_CMFD_V0106_B-01_03hr_010deg_197901.nc), where VVVV is the abbreviation of variable name, vvvv is the data version, B-01 is the code of this data type, TTTT is the temporal resolution with units, SSS is the spatial resolution (with the decimal point omitted), YYYY is the four-digit year, and MM is the two-digit month.

Also, stored in directory Data_ancillary is an ancillary file named elev_CMFD_V0106_B-01_010deg.nc, which is the file for terrain elevation of the grid cells.

## Technical Validation

### Validation of the algorithms in eastern China

**Fig. 3:**
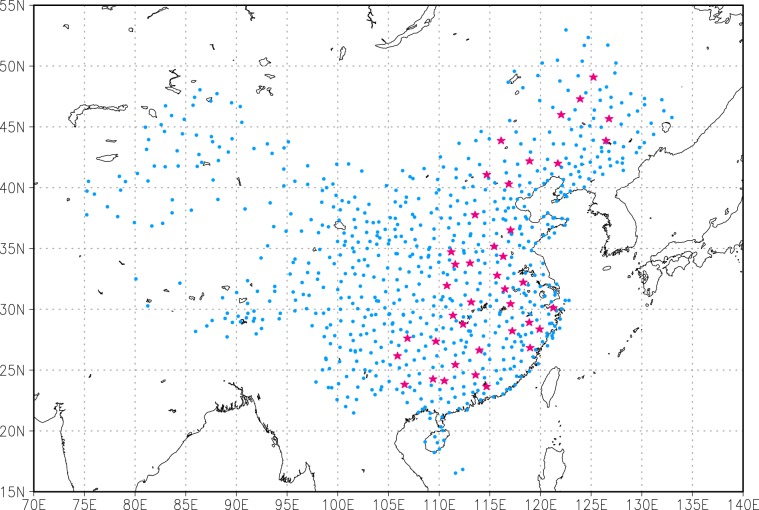
The distribution of the 753 CMA stations that supply observational data for the CMFD. The 40 magenta stars represent the stations that were removed to create a special version of the CMFD (CMFD-S) for test purposes.

The goal of the algorithm is to correct systematic biases in gridded background data using observational data. The most important question is whether the outcome of the algorithm is better than the input gridded background datasets (e.g. TRMM and GLDAS), which are widely used. To test the capability of the algorithm, we removed observation data of 40 stations in eastern China from the 753 stations that used to create the CMFD (Fig. 3), and then reran the code. This gave us a special version of the CMFD (hereafter referred to as CMFD-S, where “S” is for special, note this is not the released version of the CMFD). Because the observational data of the 40 removed stations are not involved in creating the CMFD-S, they could be used as independent observations to evaluate the result of the algorithm, the CMFD-S.

**Fig. 4:**
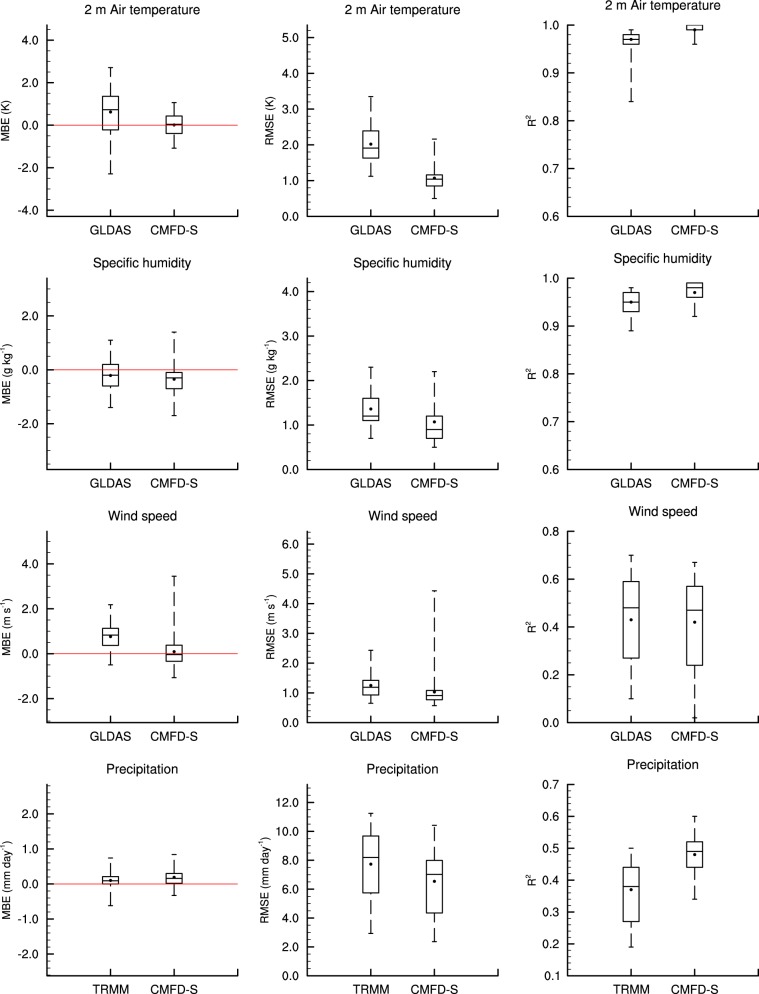
Statistical error metrics of the CMFD-S based on evaluation at the removed 40 stations compared with the gridded background (GLDAS NOAH10SUBP 3H or TRMM 3B42) dataset, for the period of 2001–2010. Panels from left to right in each row are the mean bias error (MBE), root mean square error (RMSE) and coefficient of determination (R^2^), respectively. All the error metrics are calculated on daily scale. The top and bottom boundaries of a box are the upper and lower quartiles of the statistic indices at these stations, while the line inside the box is the median. The vertical dashed lines extending from the box represent the minimum and maximum of the corresponding indices. Additionally, the dots denote the mean values of the indices. The red line in each left panel denotes MBE = 0.

The mean bias error (MBE), root mean square error (RMSE), and coefficient of determination (R^2^) of both the background data and the CMFD-S on a daily time scale were calculated for each of the 40 stations, and the results are summarized as Fig. 4. For temperature and specific humidity, the CMFD-S has lower RMSE and higher R^2^ than GLDAS, which serves as the input gridded data for creating CMFD-S. The MBE of the CMFD-S air temperature is clearly less than that of GLDAS, and the MBE of CMFD-S specific humidity is similar to that of GLDAS. For wind speed, we can see improvement for the MBE and RMSE, but correlation is not improved, as wind speed highly depends on very local terrain. The precipitation results for the CMFD-S are only slightly improved upon TRMM data over 2001–2010. However, CMFD-S precipitation is clearly better than GLDAS over 1988–1997 (Table 2), when GLDAS serves as the gridded background before TRMM data is available. The downward shortwave and longwave radiation were not evaluated because they are not routinely observed by CMA weather stations. The surface pressure data was neither evaluated in eastern China, because (1) the variability of surface pressure is less important than other variables from the perspective of land surface modelling, and (2) abundant observational data are available in eastern China for surface pressure. Instead, these three variables of downward shortwave and longwave radiation, and surface pressure were validated at research stations in western China (see next section “Validation of CMFD in western China”). To conclude, the algorithm for creating CMFD is indeed capable of reducing the biases in input gridded data.

**Table 2:** Statistical error metrics of daily precipitation for the CMFD-S based on evaluation at the 40 removed stations compared with the GLDAS NOAH10SUBP 3H dataset, for the period of 1988–1997 and 1998–2007.

Period	MBE (mm day^−1^)		RMSE (mm day^−1^)		R^2^	
	GLDAS	CMFD-S	GLDAS	CMFD-S	GLDAS	CMFD-S
1988–1997	−0.27	−0.02	8.15	6.54	0.23	0.47
1998–2007	−0.04	0.13	7.16	6.64	0.39	0.48

### Validation of CMFD in western China

**Fig. 5:**
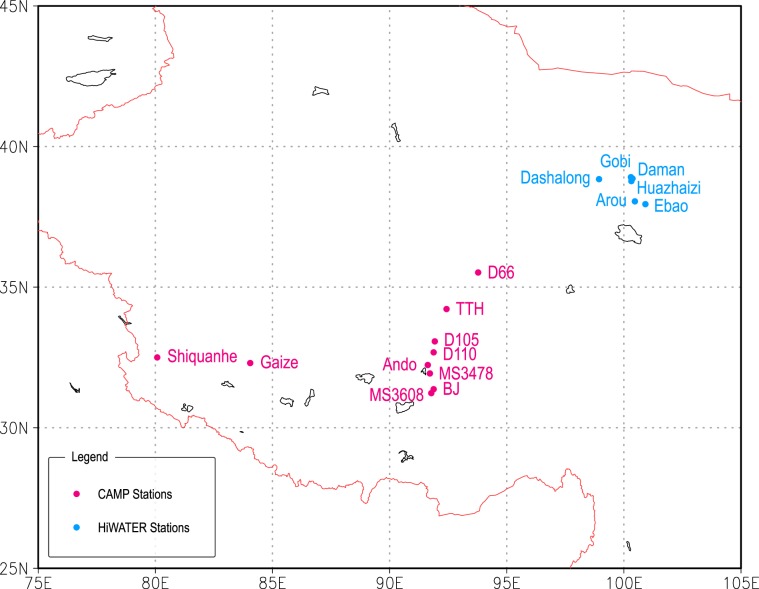
The distribution of stations in the CAMP (magenta) and HiWATER (blue) datasets.

**Fig. 6:**
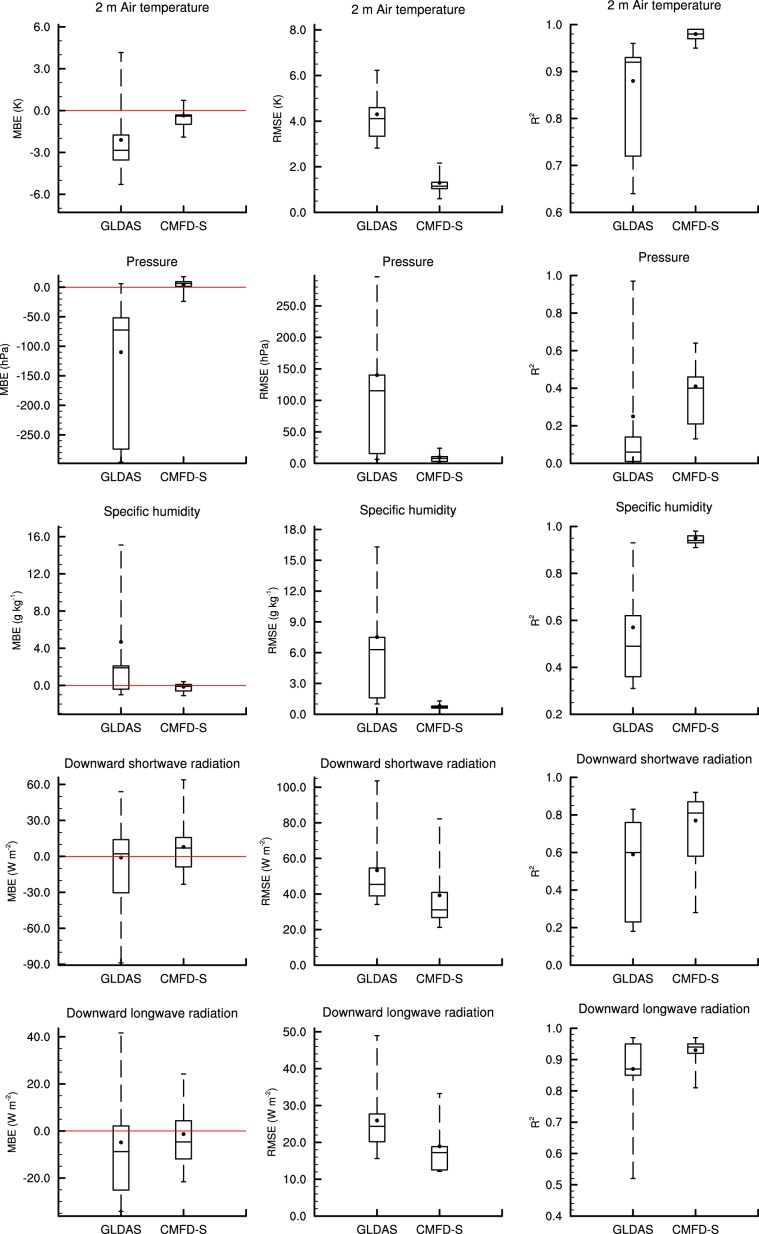
Similar to Fig. 4, but for validation results of the CMFD compared with the GLDAS NOAH10SUBP 3H dataset, using HiWATER and CAMP observations as references.

Creating the CMFD for western China where stations are sparse is another challenge. Here we use independent data to evaluate the CMFD’s performance in this region. Daily mean observation data from the Heihe Watershed Allied Telemetry Experimental Research (HiWATER)^26,27^ and the Coordinated Enhanced Observing Period (CEOP) Asia-Australia Monsoon Project (CAMP)^28^ are used to evaluate the CMFD. The distribution of stations is shown in Fig. 5. Error metrics of five variables, air temperature, pressure, specific humidity, shortwave and longwave radiation, are summarized as a boxplot (Fig. 6).

Figure 6 shows that the CMFD have closer-to-zero MBE, lower RMSE and higher R^2^ than GLDAS for almost all variables. In other words, according to statistical indices, the CMFD is generally better than GLDAS in regions where weather stations are sparse.

## Usage Notes

This dataset saves space by having packed 32-bit float values into 16-bit short integers, thus, these data need to be unpacked before use. Most high-level software (GrADS, Matlab, etc.) can automatically unpack this kind of data in the background so users do not need to do this themselves. However, when users want to write FORTRAN or C programs to read this data, the following formula should be used to restore 32-bit float data from 16-bit short integers:

$$32 \mbox{-} {\rm{bit}}\_{\rm{unpacked}}\_{\rm{data}}\_{\rm{value}}=16 \mbox{-} {\rm{bit}}\_{\rm{packed}}\_{\rm{data}}\_{\rm{value}}\,\ast \,{\rm{scale}}\_{\rm{factor}}+{\rm{add}}\_{\rm{offset}},$$

where the scale_factor and add_offset are two parameters needed for unpacking the data. Table 3 lists the scale_factor and add_offset for each variable.

**Table 3:** Scale_factor and add_offset for each variable in the CMFD.

Variables	Variable name	Scale_factor	Add_offset
Temperature	temp	0.01	273.15
Pressure	pres	2.00	63500.00
Specific humidity	shum	0.000001	0.025
Wind speed	wind	0.002	60.00
Downward shortwave radiation	srad	0.25	685.00
Downward longwave radiation	lrad	0.25	685.00
Precipitation rate	prec	0.0025	50.00

A simple FORTRAN program named PRG-01.01_Data_Read_Example.f90 is provided along with the dataset as a sample to show users how to read the NetCDF data files, but users will most likely need modify it to meet their particular demands. This program has been tested on both Linux and Windows platforms with netcdf-3.x libraries, and anyone who wants to compile this program must have the NetCDF library installed first. For more information about NetCDF, users may refer to https://www.unidata.ucar.edu/software/netcdf/.

## Data Availability

The code used in this work is not published along with the dataset because a non-free software named ANUSPLIN is invoked by this code, and cannot be redistributed without permission.
